# Unravelling Heterozygosity-Rich Regions in the Holstein Genome

**DOI:** 10.3390/ani15152320

**Published:** 2025-08-07

**Authors:** Michael Smaragdov

**Affiliations:** Russian Research Institute of Farm Animal Genetics and Breeding (RRIFAGB), Branch of the L.K. Ernst Federal Science Center for Animal Husbandry, St. Petersburg 196601, Russia; mik7252@yandex.ru

**Keywords:** heterozygosity, heterozygosity islands, herds, cattle

## Abstract

Genomes of animals harbor not only homozygous (ROH) but also HRR segments of chromosomes. In the current study, the HRRs in Holstein cattle were analyzed using a 50K array. On average, 366 Holstein cows from six herds had 439 ± 7 and 111 ± 3 HRRs per cow when HRRs with a minimum length of 50 kb and 250 kb were detected, respectively. The average HRRs values did not differ between herds. Removal of SNP with an MAF less than 0.01 leads to a noticeable increase in the number of HRRs. The distribution of HRRs in the chromosomes of cows is not uniform and depends on the scanned lengths of the HRR segments. No clear correlations were found between interchromosomal HRR specific density and the occurrence of HRRIs. When HRR segments with a length of 50 kb or more have been detected, the significant HRRIs were located on BTA1, BTA10, BTA20, and BTA21(first set); for HRR segments with a length of 250 kb or more, significant islands were found on BTA9, BTA11, and BTA29 (second set). According to the Tajima D test, the islands on the BTA of the first set have maximum values of 3.47–2.82, while the islands on the BTA of the second set have values of less than 2.49. The genes in the HRRIs are involved in immune response, oogenesis, and reproduction but are not related to milk production traits. These results show that the recognition of HRRIs strongly depends on the length of the scanned HRR segments. No single “mechanism” was identified as the main cause of HRRIs.

## Simple Summary

The quantitative characteristics, the distribution on the chromosomes, and above all, the possible functional role of the heterozygote-rich regions (HRRs) in the animal genome have not yet been clarified. Some characteristics of HRRs and HRR islands (HRRIs) in the genome of Holstein cows from six herds were analyzed. The herds do not differ in the average number of HRRs. The removal of SNPs with a minor frequency (MAF) of less than 0.01 leads to a significant increase in the number of HRRs. The HRRs are not evenly distributed across the chromosomes. The localization of the HRRIs on the chromosomes depends on the minimum length of the detected HRRs. Overall, the Tajima D test partially confirms the occurrence of significant HRRIs due to balanced selection. In addition, possible causes such as recombination, inversions, and mobile elements that may contribute to the occurrence of HRRIs are discussed.

## 1. Introduction

Since genetics emerged as a science, homozygosity and heterozygosity have been widely used to evaluate the genome of animals. In the last decade, thanks to the concept of runs of homozygosity (ROH), extended autozygous loci in the genomes of animals have been intensively studied [1,2]. For example, ROH-based autozygosity analysis has helped to understand the nature of inbreeding depression [3,4]. However, heterozygosity-rich regions (HRRs) have not been characterized as comprehensively as ROH, and the concept of HRR in animal genomes was first introduced, operationally defined, and critically discussed by Ferenčaković et al. [5]. Other synonymous terms for the same phenomenon have been proposed in the literature ROHet [6] and HER [7]. Based on the pioneering study by Williams et al. [8] and Ferencakovic et al. [5], who found HRR clusters as large as 150 kb in the genome of semi-wild Chillingham cattle and HRRs of 58 kb–1.4 Mb in Austrian Pinzgauer bulls, respectively, research is currently underway to identify HRRs in other animals. HRRs have been detected in turkeys [9], a local horse breed [10], two Duroc pig populations [11], sheep populations around the world [12], in the genomes of commercial and local goat breeds [13], in 30 Italian and 19 worldwide goat breeds [14], in five Alpine and Mediterranean goat breeds [15], in goats from four farms in Greece [16], in canine genomes [17], in the Italian Simmental cattle breed [6], in semi-wild Maremmana cattle [7], in Holstein cattle [18], and in cosmopolitan and local Italian beef cattle breeds [19].

Another phenomenon discovered during the study of HRRs, namely their concentration in certain regions of the chromosomes, the so-called HRRI, deserves special investigation. We need to find out whether this phenomenon has a biological explanation. All the studies cited above suggest that the cause of the occurrence of HRRIs is balanced selection [20,21], which leads to the heterozygosity of some alleles of genes in HRRIs. Therefore, all studies have focused on the identification of genes in HRRIs. Starting with the studies of Williams et al. [8] on Chillingham cattle and Ferencakovic et al. [5] on Austrian Pinzgauer cattle, attempts have been made to identify genes in HRRIs and their functions. Is balanced selection the only cause for the emergence of HRRIs? Other factors such as high recombination rate, inversions, and variable structural elements (e.g., SINE and LINE) can lead to HRRI occurrence. The listed hypotheses were tested in this study. For this task, the Holstein breed was selected, as the best-studied breed in the world, which plays a prominent role in the production of dairy products.

This study should be seen as an extension of previous findings by characterizing HRRs across multiple cattle populations that have not been extensively studied [5,6,7,8,18,19,22]. The aim of this study was to obtain data on regions enriched with HRRs in Holstein cows, including the number and length of HRR segments as well as their distribution on the chromosomes. The effects of SNPs with an MAF < 0.01 on the number of HRRs were also tested. Particular attention was paid to HRRIs and the genes within these islands. Causes that could lead to the occurrence of the phenomenon of HRRIs are discussed based on our own data and date from the literature.

## 2. Materials and Methods

### 2.1. Animal Resources and SNP Genotyping

The Holstein cows analyzed in this study were born between 2010 and 2013 in six herds in the Leningrad Region (Russia). For more information on the breeding of Holstein cattle in the Leningrad Region, see this article [23]. The animals for genotyping were selected by the farmers, taking into account the pedigree structure of the herd. The animals sampled represented 8–15% of the total number of dairy cows in the herds. To identify the putative diversity of HRRs in Holstein herds relevant to the objectives of the research, data on genetic distances between 12 herds using a fixation index and principal component analysis were used, which were previously determined [24]. Among them, the six most distinct breeds according to these criteria were selected. A total of 366 cows from six herds were genotyped using the Bovine SNP50 v.2.0 array (Illumina, San Diego, CA, USA). Quality control was performed using Plink v. 1.9 [25]. (i) SNP calls with a quality score of less than 0.7 were removed. (ii) Only autosomal chromosomes were considered. (iii) The comands geno 0.05 and mind 0.05 were applied, resulting in 48,108 SNPs for cows. When SNPs were removed with an MAF < 0.01, the total number of SNPs was 43,399. The overall genotyping rate was >0.99.

### 2.2. Identification of HRRs and ROHs

HRR and ROH segments were identified using the R package detectRUNS 0.9.5 [26]. The parameters for the method of consecutive runs for HRR were as follows: (i) the minimum number of SNPs included in the HRRs was 5; (ii) the number of homozygous SNP calls allowed in HRR segments was set to zero; (iii) the minimum length of HRR segments was set to 50 kb or 250 kb; (iv) the maximum distance between SNPs was set to 1 Mb.

The parameters for the consecutive run method for ROH were as follows: (i) the minimum number of SNPs included in the ROHs was 10; (ii) the number of heterozygous SNP calls allowed in ROH segments was set to zero; (iii) the minimum length of HRR segments was set to 250 kb; (iv) the maximum distance between SNPs was set to 1 Mb.

### 2.3. Calculation of HRRIs Significance

The significance of the identified HRRI was not determined in the published articles. Instead, restrictions were placed on the selection of HRRIs depending on whether the HRRIs were identified by selecting SNPs with a frequency in the HRR > a certain value [7] or by extracting the 99.9 quantile of the SNP frequency distribution within the HRRs [27]. To assess the significance of HRRIs, we made the following assumptions: (i) each herd has its own combination of HRRIs; (ii) each HRRI has its own proportion of HRRs in the herd; (iii) if we merge the herds into one dataset and perform a permutation of the cow genomes, we obtain the distribution of the mixed cow genomes; (iv) by comparing the proportion of HRRs in HRRIs from a herd with the proportion of HRRs in HRRIs from the mixed distribution, it is possible to estimate by how much the proportion of HRRs in HRRIs from a herd exceeds the proportion of HRRs in HRRIs in the mixed distribution. The mixed distribution was developed according to the following procedure.

The PED files of cows from 1 to 6 herds were included in the dataset. Each PED file of a cow was assigned a pseudo-phenotype in the sixth column, which corresponds to the cow number from 1 to 366 in the data set (1, 2.1, 3, -8, 9.1, -366). The numbers 2 and 9 have their own program meaning in PLINK; thus, you cannot write 2 and 9. PLINK then permuted these pseudo-phenotypes and the PED files labeled with them accordingly. This pseudo-phenotype serves as a label for the PED file of the respective cow. If we rearrange the pseudo-phenotypes, we also rearrange the PED files of the cows in the mixed data set. The pseudo-phenotypes in the PED files of the mixed dataset of all six herds were permuted 10,000 times using the --make-perm-pheno command in Plink 1.9. This command resulted in all cow PED files being rearranged 10,000 times. The permuted dataset was divided into six groups of sixty cows each (simulated six permuted herds), and the HRRIs were identified for each group. Among these, HRRIs whose localization matched the HRRIs previously identified for six herds were selected. Then, for the HRRIs of six permuted herds that were identical to each HRRI in the six herds, the mean and error were calculated based on the proportion of HRRs in each permuted HRRI. The significance of each HRRI in the six herds was calculated using the Mann–Whitney U test by comparing the proportion of HRRs in the HRRs for the six permuted herds with the proportion of HRRs in the herd HRRIs.

### 2.4. Tajima D Test

The Tajima D test was carried out using the VCFtools software v.0.1.13 [28]. Genome scanning was performed using a 100 kb frame. The size of this frame was chosen based on the length of the HRRIs, which ranged from 50 kb to 500 kb. The SD value was calculated for each chromosome in the herd in which a significant HRRI was identified. The SD value was derived from the entire set of Tajima D values on the chromosome. The threshold value of 3 SD is often used to eliminate outliers. Tajima D values of less than 3 SD were considered outliers. Sensitivity to demographic history was not considered.

### 2.5. Chromosome Rank Calculation and Coefficient of Extended HRRs

A ranking order was proposed to systematize the chromosomes according to the specific density of their filling of HRRs. The rank of chromosomes based on the number of HRR in them was calculated using the following formula: HRR rank of a BTA_i_ = Ni/TBTAi sizecattle genome size , where N_i_ is the number of HRRs on a BTA_i_, and T is the total number of all HRRs on 29 autosomes.

The total length of the ROH segments mostly was used to calculate the inbreeding coefficient [2]. By analogy, the total length of HRR segments in the cow genome can be used to calculate their proportion in the cow genome, which is why we have labeled it the coefficient of extended heterozygosity. This coefficient can serve as a measure of the prevalence of HRR segments in the animal’s genome. The coefficient of extended heterozygosity for HRRs was calculated as the length of all HRR segments in autosomes divided by the size of all autosomes covered by SNP for each cow. They were then averaged for all cows across the herds and across the entire data set.

### 2.6. Genes, SINE, and LINE Element Annotation

The locations of genes within HRRIs were retrieved from the National Center for Biotechnology Information (NCBI) (https://www.ncbi.nlm.nih.gov/gdv/browser/genome, accessed on 3 August 2025). The reference assembly of the bovine genome ARS–UCD 2.0 was used. The SINE, LINE, Simple Repeats, and LTR elements were retrieved from the ROH islands and HRRIs using the Genome Browser UCSC [29].

## 3. Results

### 3.1. Description of Genetic Differences Between Herds

Genetic variation is the basis for the survival and maintenance of cattle populations. At the genome level, variation occurs in the form of considerable allelic diversity and heterozygosity. It is well known that selection promotes inbreeding. Can artificial selection lead to chromosomal regions enriched in heterozygosity? To answer this question, herds must be selected with cows that differ in their allelic diversity. The allelic differences of SNPs between Holstein herds are listed in the article [23]. Briefly, pairwise differences between six herds, as measured by the fixation index (Fst), ranged from 0.003 to 0.12 and were all significant (*p* ≤ 1.28 × 10^−32^). The fourth breed differed the most from the other five breeds, by 0.006, 0.012, 0.009, 0.009, and 0.004. Principal component analysis confirmed the results obtained with Fst. Thus, differences were found between six breeds based on Fst and principal components, with breed 4 differing the most from the others. It needs to be verified whether the observed allele frequency differences between herds are due to HRRs or not.

### 3.2. Identification of HRRs

Preliminary scanning of the cow genome showed that the majority of detected HRR segments range from 50 kb to 2.0 Mb. It was found that the optimal number of 5 heterozygous consecutive SNPs is required to effectively define the length and number of HRRs. When larger values (10–15) were used, the number of HRRs was significantly lower. The descriptive HRR statistics are shown in Table 1 and Table 2. The mean number of HRR segments across all breeds was 335 ± 6 when 50 kb HRRs were used as minimum scanned HRR segments (first variant) (Table 1) and 111 ± 3 when 250 kb HRRs were used as minimum scanned HRR segments (second variant) (Table 2). No significant differences were found between the mean numbers of HRR segments in the six herds in both variants (*t*-test).

The effect of SNPs with an MAF < 0.01 on the number of HRRs was also tested. When SNPs with an MAF less than 0.01 were removed, the average value of HRRs was 439 ± 7 in the first variant (Table 1) and 200 ± 3 in the second variant (Table 2). Thus, the removal of these SNPs from the cow genome leads to an increase in the number of HRR segments, indicating the homozygosity of the removed SNPs that separated the heterozygous segments.

**Figure 1:**
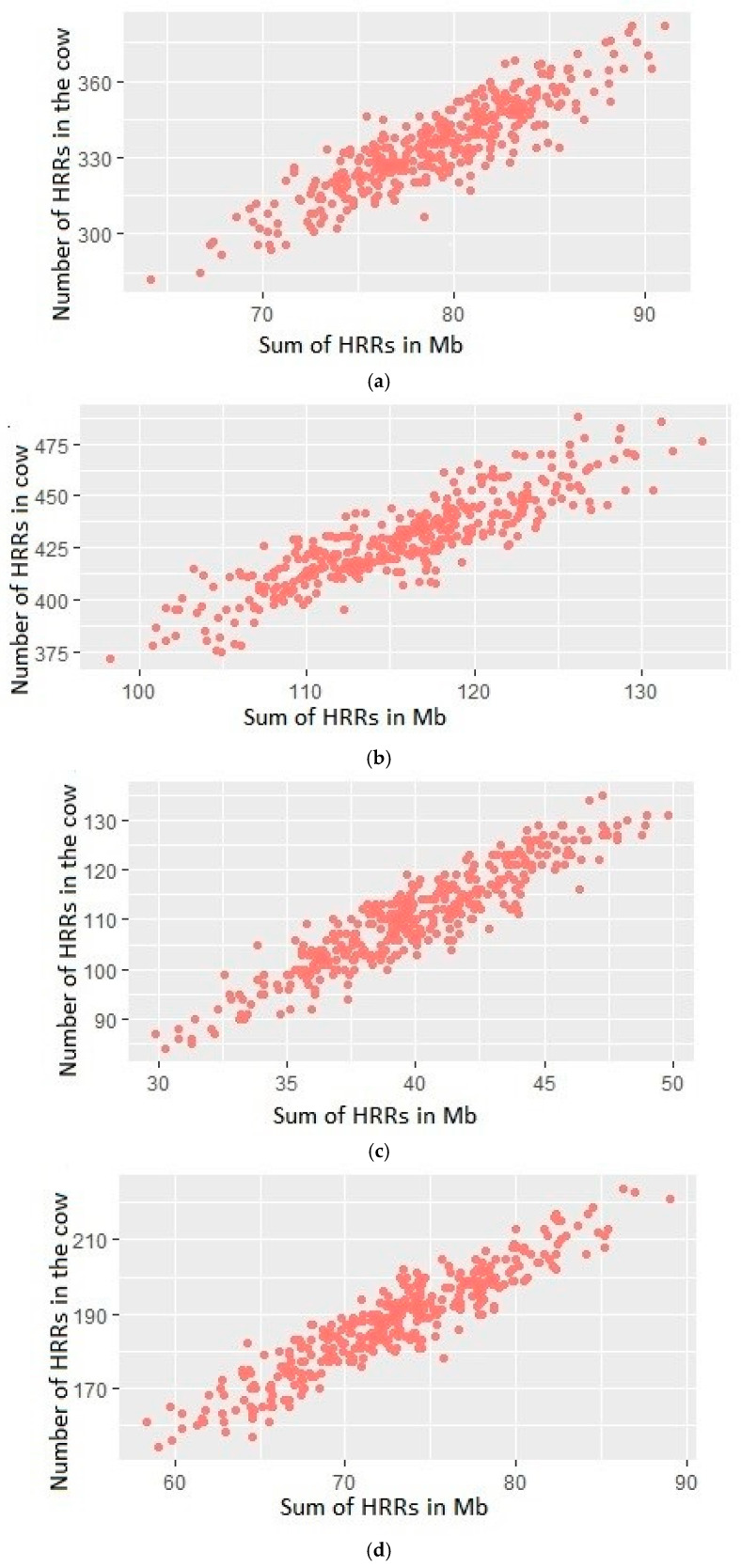
Distribution of the total length of HRRs vs. number of HRRs per cow. The number of cows is 366. (**a**) Set of SNP with all MAFs. The minimum length of HRR segments is 50 kb. (**b**) SNPs with an MAF less than 0.01 were removed. The minimum length of HRR segments is 50 kb. (**c**) Set of SNP with all MAFs. The minimum length of HRR segments is 250 kb. (**d**) SNPs with an MAF less than 0.01 were removed. The minimum length of HRR segments is 250 k.

Figure 1 shows the dependence of the total size of HRRs in the cow genome on the number of HRRs per cow for the first and second variants and when SNPs with an MAF < 0.01 were removed. In both variants, an increase in the number of HRR segments is observed when SNPs with an MAF of less than 0.01 are removed. In the cow genome, an almost linear dependence of the number of HRRs on their total length is observed. This dependence indicates low diversity in HRR lengths in Holstein cows.

To estimate the variation in HRR lengths, their lengths were divided into five classes. With a minimum scanned HRR length of 50 kb, most HRRs were between 50 kb and 0.8 Mb in length, and very few HRRs were over 1.6 Mb in size (Table 3). When SNPs with an MAF of less than 0.01 were removed, the number of HRRs with a length from 200 kb to 1.6 Mb mainly increased, while HRRs with a length of 50 kb to 200 kb increased slightly, i.e., short HRR segments up to 200 kb were clustered. When scanning HRR segments longer than 250 kb, their maximum number was between 250 kb and 800 kb (Table 4). When SNPs with an MAF of less than 0.01 were removed, the number of HRR segments increased in all classes, indicating that they were mainly formed from clustered HRR segments less than 250 kb in length. The coefficient of extended heterozygosity, calculated as the sum of the lengths of all HRRs in chromosomes divided by the total length of the bovine chromosomes covered by the SNP, is equal to 0.0289 ± 0.0001 (for HRR lengths greater than 250 kb) and 0.0315 ± 0.0001 (for HRR lengths greater than 50 kb). The coefficient of inbreeding is equal to 0.111 ± 0.003 [30]. Thus, the total length of the HRR segments is five times less than the total length of the ROH segments. Accordingly, the selection of Holstein cattle had no significant effect on the formation of HRRs.

The rank of HRRs in the bovine chromosomes was calculated considering the relative length of each chromosome in the bovine genome. For each chromosome, the HRR rank was calculated as the proportion of its HRR segments in the total number of HRR segments in all chromosomes divided by the proportion of its size in bovine chromosomes (see Section 2) (Table 5). This parameter characterizes the specific density of the distribution of HRR segments in the chromosomes of cows. The chromosome rank distribution changes when SNPs with an MAF of less than 0.01 were removed for both scan variants (50 kb and 250 kb) (Table 5 and Table 6). This fact indicates an uneven distribution of homozygous SNPs removed between the HRR segments in the cow chromosomes.

The current study is the first report to elucidate the distribution of HRRs’ specific densities across all chromosomes of Holstein cows. Of the 29 chromosomes, BTA14, BTA25, and BTA6 (and BTA14, BTA13, and BTA6 after removal of SNP with an MAF < 0.01) have the highest HRR specific density, while BTA24, BTA22, and BTA12 (and BTA23, BTA27, and BTA9 after removal of SNP with an MAF < 0.01) have the lowest HRR specific density with a minimum scan length of HRRs greater than 50 kb (first variant) (Table 5). When the minimum HRR scan length exceeded 250 kb and after removal of SNP with an MAF < 0.01, the chromosome distribution changed slightly—BTA14, BTA25, and BTA5 (and BTA14, BTA5, and BTA13) had the highest HRR specific density, and BTA20, BTA12, and BTA22 (and BTA28, BTA20, and BTA27 had the lowest HRR specific density (second variant) (Table 6).

For the first variant, the Pearson and Spearman correlations between the percentage of HRRs in the chromosomes and the relative length of each chromosome in the bovine genome were not significant (*p* = 0.87 and *p* = 0.60, respectively), with a correlation coefficient of r < 0.3. For the second variant, these values were *p* = 0.58 and *p* = 0.20, with a correlation coefficient of r < 0.25 when SNPs with an MAF < 0.01 were not removed. If SNPs with an MAF < 0.01 were removed, then the Spearman correlation was 0.43 (*p* = 0.02). The correlation indicates a general trend to fill chromosomes with HRR segments, while chromosome rank more accurately calculates the actual density of HRR segments in each chromosome. Of all chromosomes, BTA14 has the highest rank in Table 5 and Table 6, which differs from the mean rank value by more than two standard deviations, with a significance of *p* < 0.01. (Mann–Whitney U test). Thus, there is a deviation from the uniform distribution of HRR segments on 29 chromosomes, except for the Spearman correlation for the 250 kb variant when MAFs < 0.01 were removed (*r* = 0.4, *p* < 0.02). It should be noted that, despite the increased saturation of BTA14 with HRRs, no HRRIs were found on this chromosome (see below).

### 3.3. Identification of HRRIs and Annotation of Genes Within Them

Using the permuted distribution of the cows’ genome allowed us to find significant HRRIs. These were the HRRIs with the largest proportions of HRRs (Tables S1–S4). The last columns of these tables show the proportion of HRRs in HRRIs detected in 366 cows in six herds (complete set). These data were obtained by merging all cows in one set. Several HRRIs were found in the herds that were not found in either the six permuted sets or the full set. For some islands, only one or two HRRI variants were found in the six permuted sets (Tables S1–S4). Therefore, the significance of these HRRIs could not be assessed, and they were marked as not evaluable. All were tested for the hypothesized effect of balanced selection using the Tajima D test (Tables S1–S4). The significance of the difference between the HRR proportion value for each HRRI in the six herds and the proportion values in the six permuted groups for the same HRRI was calculated using the Mann–Whitney U test. The results for the significantly (*p* < 0.05) identified HRRIs are shown in Table 7.

As already mentioned, the removal of SNPs with an MAF of less than 0.01 leads to the removal of homozygous (mostly monomorphic) SNPs and rare alleles. Theoretically, these SNPs may not only be noise but also traces of positive selection for some SNPs. When scanning was performed with a minimum HRR length of 50 kb, nine dissimilar islands were found among all HRRIs after the removal of SNPs with an MAF < 0.01 (Table S2). Only one of them was significant in BTA1 (103.6–103.7 Mb). It has a low Tajima D value (Table 8). Thus, in this case, the removal of monomorphic SNPs and rare SNPs did not result in a significant change in HRRI score, suggesting that the removed SNPs may be related to directional rather than balanced selection.

When scanning was performed with a minimum HRR length of 250 kb, ten dissimilar HRRIs were found among all HRRIs after the removal of SNPs with an MAF < 0.01 (Table S4). Only one of them was significant in BTA11 (34.2–343.4 Mb), while five had a Tajima-D value < 3 SD, and one in BTA12 (41.6–42.0 Mb) had a Tajima D value > 3 SD. In this case, the removal of the monomorphic SNPs also did not lead to a significant change in the HRRIs score. Overall, with a minimum scanned HRR of 50 kb, five significant HRRIs with maximum or close to maximum Tajima D values were identified, whereas, with a minimum scanned HRR fragment of 250 kb, only three significant HRRIs with Tajima D values of less than 3 SD were found. Therefore, for 50K arrays, it is desirable to scan genomes with minimum HRR values of 50 kb and 250 kb.

The length of HRRIs ranged from 51.1 to 255.6 kb when the minimum scan length was 50 kb. Two HRRIs were detected on BTA1 in the regions of 66.48–66.66 Mb and 103.67–103.72 Mb. The second HRRI region was identified in herd 1, provided SNPs with an MAF < 0.01 were removed. No genes were found in this HRRI. The first HRRI was identified in herds 1, 2, and 4 when SNPs with all MAFs were used and in the same herds when SNPs with an MAF < 0.01 were removed. The following three genes are located in this HRRI: (immunoglobulin-like domain-containing receptor 1) *ILDR1*, (CD86 molecule) *CD86*, and (calcium-sensing receptor) *CASR*. *ILDR1* is a gene related to water transport and affects the mechanisms of paracellular water transport and urine concentration. Overexpression of this gene significantly reduces paracellular water permeability [31]. This gene had the most significant effects on pregnancy rate and cow conception rate in Holstein cattle [32]. The *CD86* gene is required for T cell activation [33] and is also involved in the determination of the innate immune mechanism of the early embryo in dairy cows [34]. The *CASR* gene is involved in the maintenance of Ca^2+^ homeostasis in the blood and has been reported to have been introduced into East Asian Indian cattle from wild cattle species [35].

The *ZNF609* gene (zinc finger protein 609) was localized in the HRRI on BTA10 in the region 45.46–45.56 Mb in herds 1 and 4. In the Asturiana de los Valles cattle breed, the *ZNF609* gene has been proposed as a candidate gene involved in myogenesis [36]. circRNA ZNF609 derived from the *ZNF609* gene sequence has been shown to be involved in the development and progression of many diseases [37].

On BTA20, HRRI was identified in the region of 40.83–40.98 Mb in breed 3 (Table 7). The (natriuretic peptide receptor 3) *NPR3* gene was localized in this HRRI. It was shown that normal cumulus expansion in bovine cumulus–oocyte complexes is associated with an inhibitory effect of gonadotropins on *NPR3* mRNA expression, which is mediated via the epidermal growth factor receptor. The study also provides evidence that CNP and NPR3 synergistically interact to regulate the expansion of the cumulus in response to gonadotropins [38].

On BTA21, HRRI was identified in the region of 2.93–2.98 Mb in breed 4 (Table 7). The gene (ATPase phospholipid transporting 10A (putative)) *ATP10A* was localized in this HRRI. *ATP10A* is a transmembrane protein of the p-type cation transport ATPase family [39]. P0910 and OmpH were also found to activate the expression of several genes, including *ATP10A*, upon infection with Pasteurella multocida in yak [40], and to be associated with female reproductive performance in Qaidam cattle [41]. This gene was placed in HRRI on BTA21 in the region of 2.1–3.2 Mb in Italian Simmental cattle [6].

By scoring HRR segments, starting with the smallest size of 250 kb, the HRRIs listed in Table 7 were identified. On BTA29, a significant HRRI was found in the region of 40.02–40.28 Mb in herds 1, 4, and 5. This HRRI includes several LncRNA, and the genes (synaptotagmin 7) *SYT7*, (diacylglycerol lipase alpha) *DAGLA*, (myelin regulatory factor), *MYRF*, and microRNA mir-2885 were also localized. The SYT7 gene may be associated with temperament in sheep [42]. In humans, the methylation level of *SYT7* has been significantly associated with hypertension [43]. It has been suggested that *SYT7* is subject to balanced selection in the bovine genome [44]. In the gastrointestinal tract of cattle, endocannabinoid synthesis probably plays a comparable role in mediating inflammation in the epithelium. The *DAGLA* gene is involved in the endocannabinoid system in bovine tissues [45]. The *MYRF* gene is a master regulator governing myelin formation and maintenance in the central nervous system. The conservation of *MYRF* across metazoans and its broad tissue expression suggest that it has functions extending beyond the well-established role in myelination [46]. The *MYRF* gene is a potential candidate as an early regulator of gonadal development in sheep via upregulation of the transcriptional cofactor CITED2 [47].

When scanning HRRs over 250 kb without MAF restriction, the HRRI was significantly identified on BTA9 in the region of 43.94–44.32 Mb (Table 7). Several LncRNAs and pseudo genes of the large ribosomal subunit protein, eL6-like and S25, are located in this region.

On BTA11, HRRI was identified in the region of 34.20–34.48 Mb in breed 2, when SNPs with an MAF < 0.01 were removed (Table 7). No protein coding genes were found in this HRRI except for several LncRNAs. It is worth noting that, when HRR segments longer than 250 kb were scanned, the length of the HRRI regions increased, and these regions in BTA9 and BTA11 lack genes encoding proteins. Such heterozygosity-rich regions may have arisen due to the lack of selection pressure in these regions.

In summary, the *LDR1* and *ATP10A* genes involved in reproduction, *CS86* in immunity, *NRP3* in oogenesis, and *SYT7* may be subject to balanced selection. We can conclude that no genes associated with milk yield and composition of cows were found in these HRRIs.

To test whether the HRRIs are confirmed as traces of balanced selection, the Tajima D test was applied [48]. Of the eight significant HRRIs (Table 8), four (BTA1 (66.5–66.6 Mb), BTA10 (45.3–45.4 Mb), BTA20 (40.7–40.8 Mb), and BTA21 (2.8–2.9 Mb)) had maximum Tajima D values or were close to the maximum D value, one (BTA29 (40.0–40.1 Mb)) had more than three standard deviations, and three (BTA1 (103.6–103.7 Mb), BTA9 (43.9–44.0 Mb), and BTA11 (34.1–34.2 Mb)) had less than three standard deviations. Thus, three of the eight significant HRRIs have a low probability of being confirmed by the Tajima D test. Consequently, some HRRIs can be considered as signals of balanced selection, provided that the reliability and validity of the Tajima D test is limited. It should be recalled that Tajima D. Ref. [48] proposed an estimate of balanced selection under the assumption that the population evolves according to mutation–drift equilibrium.

### 3.4. Gene Annotation of Unevaluated HRRIs Identified by the Tajima D Test with Maximum D Values

When scanning the cow genome with a minimum HRR length of 50 kb among the 43 unevaluated HRRIs, seven had the maximum Tajima D value, 12 had Tajima D values of more than 3 SD, and 24 had Tajima D values of less than 3 SD (Tables S1 and S2). Among the unevaluated HRRIs with maximum Tajima D values were HRRIs on BTA5 (51.21–51.39 Mb and 114.29–114.50 Mb), BTA9 (94.9–95.1), BTA14 (53.17–53.37 Mb), and BTA21 (82.32–83.13 Mb). It is necessary to consider which genes are localized in these HRRIs. The genes *USP15* (ubiquitin-specific peptidase 15) and *TAFA2* (chemokine-like family member 2) are located on BTA5 in the region of 51.21–51.39 Mb. The *USP15* gene is expressed in male germ cells and is associated with the fertility traits of Nellore bulls [49]. This gene is associated with the deubiquitinating effect of proteins involved in the regulation of Parkinson’s disease, viral infections, and cancer-related signaling networks [50].

The results showed that TAFA2 directly binds to the lectin-like domain of ADGRL1 and activates the cAMP/PKA/CREB/BCL2 signaling pathway, which is critical for preventing cell death. These results suggest that *TAFA2* and its receptor *ADGRL1* are potential therapeutic targets for neurological diseases [51]. Other results show a neuroimmune interaction in which neuron-derived TAFA2 recruits CCR2+ macrophages to the liver and triggers liver injury that is at least partially reduced by nerve signaling in response to acidic stimuli, i.e., consumption of acidic substances [52].

The gene *EFCAB6* (EF-hand calcium binding domain 6) is located on BTA5 in the region of 114.29–114.50 Mb. The *EFCAB6* gene plays a crucial role in lipid metabolism and adipocyte proliferation and may contribute to Sub-Saharan breed survival in a resource-limited environment [53]. In addition, the *EFCAB6* gene is linked to spermatogenesis in admixed Swiss Fleckvieh bulls through its involvement in the androgen signaling pathway [54].

The genes *AOPEP* (aminopeptidase O (putative)) and *FANCC* (FA complementation group C) are located on BTA9 in the 94.9–95.1 Mb region. AOPEP is a proteolytic processing enzyme that is preferentially expressed in glia and may be associated with endosomal–lysosomal pathways. The autosomal recessive Zech-Boesch syndrome associated with *AOPEP* is of worldwide importance for the diagnosis of genetic dystonia [55].

In the porcine genome, the *FANCC* gene correlates significantly with features of developmental anomalies such as anal atresia [56]. This study emphasizes the complexity of the FANCC protein, with different domains responsible for DNA repair and mitophagy. Mitophagy was preserved in the *FANCC* c.67delG mutation, in contrast to the *FANCC* null mutation, which leads to an accumulation of damaged mitochondria. The results suggest that developmental defects in Fanconi anemia may not only be due to DNA repair deficits, but they may also affect other functions such as mitochondrial quality control [57].

The gene *DDHD1* (DDHD domain containing 1) is located on BTA14 in the region of 53.17–53.37 Mb. The *DDHD1* gene, which is involved in insulin and fat metabolism, can be considered as a candidate gene for abomasum displacement in Chinese Holstein cattle [58]. A significant SNP within the *DDHD1* gene was discovered in Korean Sapsaree dogs [59], which relates to the distraction index.

The gene *FAM169B* (family with sequence similarity 169 member B) is located on BTA21 in the region of 82.32.9–83.13 Mb. This protein belongs to a family of proteins characterized by their sequence similarity, indicating possible functional relationships between their members. However, without specific studies on *FAM169B*, its exact role is not yet clear. It is important to note that scanning the cow genome with a minimum HRR length of 250 kb did not reveal any unevaluated HRRIs with a maximum Tajima D value.

Thus, the gene *USP15* is associated with fertility traits in bulls and plays a role in regulating Parkinson’s disease, viral infections, and cancer-related signaling networks in humans. Gene *TAFA2* triggers liver damage. The *EFCAB6* gene is associated with spermatogenesis. The *AOPEP* gene is associated with autosomal recessive Zech-Boesch syndrome. The *FANCC* gene is responsible for DNA repair and mitophagy. The *DDHD1* gene can be regarded as a candidate for the displacement of the abomasum in cattle.

### 3.5. Possible Role of Structural Elements in the Formation of the HRRIs

One of the possible causes of the formation of heterozygote-enriched regions could be the localization of long interspersed elements (LINEs), short interspersed elements (SINEs), simple repeats, and long terminal repeats (LTR) in these regions. These elements are widely distributed in mammalian genomes and are subject to large structural variations [60]. Therefore, it was necessary to test whether HRRIs are enriched in these elements compared to ROH islands. The results are shown in Tables S5 and S6. It was found that the saturation of only LINE elements is more severe in HRRIs than in ROH islands (*p* = 0.011) (Table 9). Thus, the enrichment of the HRRIs with these elements does not differ from that of the ROH islands, except for LINE elements. Further studies are needed to confirm the results obtained, but perhaps the differences in heterozygosity of SNPs in these chromosomal regions are not due to quantitative differences in these elements but to qualitative differences (greater variability of some elements).

## 4. Discussion

### 4.1. Assessment of Heterozygosity-Rich Regions in the Studied Animals

In contrast to ROHs, HRRs are less studied in the livestock husbandry. In the current study, the average number of HRRs across all herds ranged from 439 ± 7 to 111 ± 3, depending on the length of HRRs and MAF retrieved, while herds did not differ significantly (*t*-test) (Table 1 and Table 2). While neither the number of ROH [30] nor HRR segments differed between breeds, breed 4 differed significantly from the other breeds when Wright’s fixation index and principal component analysis were used [23]. Only sires from the Netherlands were used in this herd, whereas sires from the USA, Canada, and Russia were used in other herds [61]. Thus, the extended ROH and HRR segments alone had no effect on allelic diversity between the Holstein herds. Recent studies show that the number of HRR segments varies greatly between animals. For example, the average number of HRRs per animal in the sheep genome was 139 [12], while another study found that there were only 28 HRRs per sheep [27]. In goat samples from six breeds, both commercial and local, the average number of HRRs per goat was 69.6 [13], while in Italian and world goat breeds, it was no more than 13 [14]. In Turkey, the number of HRRs did not exceed 58 [9], in horses 52 [10], and in Austrian Pinzgauer cattle 122 [5], while in semi-wild Maremmana cattle, 3 to 9 HRRs were found in the genome [7]. The cited data indicate a large variability in the amount of HRRs in the studied animals. This could be due to both selection targets and non-biological reasons, including the density of the arrays and the parameters of the software used.

In our study, most HRRs in local Holstein cattle have a length from 50 kb to 800 kb, which is much less than the length of ROHs that span up to 16 Mb or more [30]. HRR lengths were determined for several animals studied. Heterozygosity clusters of up to 150 kb have been found in highly inbred Chillingham cattle [8]. The average HRR length in the turkey genome was 470 kb [9]. In two populations of Duroc pigs, HRR lengths ranged from 1 to 4 Mb [11]. In a local horse breed, the HRR length does not exceed 2 Mb in most cases [10], and nine sheep breeds have an average HRR length of 460 kb [12], while in Greek goat breeds it is 780 kb [16]. The average length of HRRs detected was 806 kb in commercial breeds and 795 kb in local goat breeds [13], and it was not more than 500 kb in Italian and world goat breeds [14]. Thus, both the length and the number of HRR varied considerably in the animals studied. This variability is most likely due to the different breeding histories of the animals.

The current study is the first report to elucidate the specific density distribution of HRRs across all chromosomes of Holstein cows. The following conclusions can be drawn from the results. The distribution of HRRs on the chromosomes is far from uniform. Only the rank of BTA14 exceeds two standard deviations of the rank distribution shown in Table 5 and Table 6. The HRRIs detected in the chromosomes of the cows are located in the relatively HRR-saturated chromosomes BTA1, BTA10, and BTA21, as well as in the moderately HRR-saturated chromosomes BTA11, BTA20, and BTA29. It is noteworthy that the most HRR-saturated BTA14 does not show a significant HARRI, but rather an HRRI with the highest Tajima-D value (Tables S1–S4). Whether this fact is random or has a causal relationship requires further study. Therefore, it is not possible to draw a clear conclusion about the correlation between the rank of chromosomes and the location of HRRIs in chromosomes. However, the possibility that the local distribution of HRR segments in chromosomes is due to selection cannot be completely ruled out.

The ROH segments are also unevenly distributed across the chromosomes, with the highest coverage on BTA14, BTA16, BTA7, BTA26, and BTA8 and the lowest coverage on BTA18, BTA27, and BTA28 (the Pearson correlation between the proportion of ROH segments in the chromosomes and chromosome length is 0.2) [30]. Therefore, their distribution pattern might depend on artificial selection. Indeed, all ROH islands (Table S5) are located in the chromosomes that are most saturated with ROH segments. The maximum coverage of BTA14 with both ROH and HRR segments is remarkable. Whether this fact is random or causal needs further study. Other studies in cattle have not investigated the distribution of regions of high heterozygosity on chromosomes, with the exception of Williams et al. [8], who found extended tandem HRRs on the BTA1 and BTA7 of Chillingham cattle, while Ferencakovic et al. [5] observed the highest HRR coverage on BTA5 and the lowest on BTA13 in the Austrian Prinzgauer breed.

### 4.2. Towards Deciphering Possible Causes That May Lead to the Phenomenon of HRRIs

Some HRR segments form several regions in the chromosomes of cows, which are referred to as HRRIs. An HRRI is a chromosomal region where overlapping HRR segments are found in many animals. In the current study, eight significant HRRIs were identified on seven chromosomes (Table 7). Several phenomena may contribute to the occurrence of HRRIs. They could be the result of chromosomal properties such as high recombination rates, inversions, the concentration of structural elements such as SINE and LINE in HRRIs, or balancing selection for heterozygosity of some gene alleles in these regions, reduced effect of inbreeding in these regions, or a random event.

There are loci with increased recombination rates in Holstein chromosomes [62] that can be associated with HRRIs. The mean recombination rate in HRRI regions was within BTA1 (0.000212 ± 0.00002), within BTA9 (0.000329 ± 0.00002), within BTA10 (0.000214 ± 0.0000009), within BTA11 (0.00026 ± 0.000005), within BTA20 (0.000277 ± 0.000008), within BTA21 (0.000411 ± 0.00005), and within BTA29 (0.000410 ± 0.000005). These recombination rates are lower than the average rate in the Holstein genome of 0.00055 and the maximum rate of 0.0071 [62]. Thus, no HRRI regions showed an increased recombination rate above the average value.

Another cause that can lead to a heterozygous region on chromosomes is inversion [63]. Mutations can accumulate in the inverted region of the chromosome. It has been found that the size of a large number of inversions in the Holstein genome is about 900 bp [64], which is much less than the size of HRRIs. Moreover, a reduced recombination rate is observed in the HRRI regions of the genome of Holstein cows (see above). Therefore, to prove the association of inversions with HRRIs, sequence analysis for the presence of multiple micro inversions in HRRIs is required.

Assume that HRRIs are the result of balanced selection. Note that selection for favorable heterozygosity is extremely rare in animals [65]. P.W. Hedrick [65] wrote “This is not to say that some loci with heterozygosity advantage do not have important adaptive functions, but that their role in overall evolutionary change may be an unusual phenomenon rather than an important player in adaptation”. The theory predicts that there is higher genetic variation at sites closely associated with a locus that is maintained by a heterozygosity advantage over a long period of time [66]. The width of this region of expected excess polymorphism is an inverse function of effective population size and recombination rate and is generally an extremely narrow region around the selected variant [66]. If selection for heterozygote advantage is not strong, then an increase in polymorphism is not expected [67]. Simulations with structured populations have shown that recombination plays an important role in reducing drift and selection-induced linkage disequilibrium (LD) [68]. In agreement with these results, a strong linkage pattern of LD with ROHs compared to HRRs was observed in sheep populations worldwide, although many LD regions were also not found in ROH regions [12]. Therefore, it can be assumed that the selection pressure in HRRI regions of sheep is lower and recombination may be higher than in ROH regions. In Holstein cattle, the recombination rate is below the genome average (see above). According to M. Kimura’s [69] theory of molecular evolution, the observed variations may simply be neutral alleles that are in the process of drifting to ultimate fixation or loss. Thus, it currently remains difficult to clearly answer the central question of the classical/balanced debate: How much genetic variation is maintained by balancing selection in natural populations [70] and especially by artificial selection in livestock?

In addition, selection for a favorable allele or short-term selection can lead to selective sweeps [71]. It is known that some alleles of the gene for (diacylglycerol O-acyltransferase 1) *DGAT1*, which form major QTL that increase fat yield, can contribute to a decrease in milk yield [72]. It is possible that selection some time ago was for the mutant allele because it increases milk volume, whereas more recent selection in Holsteins has been for the ancestral allele because it increases milk fat [73]. Thus, the *DGAT1* gene may have been subject to artificial balancing selection over many decades. Nevertheless, there are no explicit HRRIs in the region of 603,981–614,153 bp on BTA14, where the *DGAT1* gene is located. Apparently, selection for heterozygosity in this case did not last long enough to form an HRRI. Moreover, artificial selection of quantitative traits mostly resulted in soft sweeps and smaller QTLs with a variance of less than 0.01 for each trait [71]. Therefore, large selection signatures should not be expected as a result of balancing selection (e.g., DGAT1 gene).

However, for the selective sweep 39.3–39.5 Mb on BTA29 [74] (which agrees with the HRRI for the localization measurement accuracy used), the substitution effect using the Israeli breeding index PD07 (milk fat and protein, fertility accounted for 14.3%, 43.1% and 15.2% of the index, respectively) was 0.03. Perhaps some selective sweeps are markers indicating proximity to HRRIs. In the Holstein cows in our study, one HRRI is close to this sweep at 40 Mb.

To what extent the values of Tajima D which were obtained here can be regarded as significant confirmation of the effect of balanced selection. The values obtained vary between unrepresented positive values for cattle (18), 1.8–4.5 for cattle (19), and 1.9–3.5 for cattle (Table 8) and average D values from 3.492 to 3.807 for dogs (17). These values show a wide variation. An acceptable estimate that would increase the reliability of the data obtained from Tajima D would be if it exceeds three standard deviations.

In all significant HRRIs in Table 8 and the HRRIs with the highest Tajima D value in Tables S1–S4, there were no single genes associated with milk productivity traits. They were associated with reproduction, spermatogenesis, oogenesis, immunity, induction of liver damage, responsibility for DNA repair and mitophagy, and for abomasal displacement in cattle. In Chillingham cattle, genes relevant to fitness or survival may be localized [8]. In Holstein cattle, genes in the HRRIs are associated with fertility, residual feed intake, stress sensitivity, and milk fat percentage [18]. In cosmopolitan and Italian beef cattle breeds, two genes in the HRRIs are associated with male fertility, while four genes are associated with lactation, and two other genes play a role in survival and immune response [19]. In Maremmana cattle, HRRIs harbor loci associated with general disease resistance and reproductive traits [7]. In Italian Simmental cattle, the genes in the HRRIs are associated with fitness traits, health, and reproduction [6]. Among the studies on other animals cited above, genes in HRRIs in goats are responsible for various factors such as productivity, reproduction, immune system, breed health and resistance, and environmental adaptation mechanisms [13,14,15,16]. Genes identified in common HRRIs in sheep may play a fundamental role in survival, as many of them are involved in brain integrity [12]. Even though the genomic coordinates of HRRIs and balancing selection signals did not fully overlap across all canine genomic regions, balancing selection may play a significant role in maintaining diversity in regions associated with various cancer diseases, immune response, and bone, skin, and cartilage tissue development [17]. Therefore, a wide range of functions can be observed in the genes identified in HRRIs.

What insights have been gained that contribute to understanding the role of HRRIs in animals? A genome-wide association analysis with HRRIs was performed in Duroc pigs, revealing the association between fitness, reproductive traits, and 33 HRRIs [11]. The annotated genes located within or near the identified HRRIs in Mediterranean domestic sheep were mainly associated with immune response, protection against livestock diseases, and other performance traits [27]. It should be noted that associative data are necessary but not sufficient evidence for gene selection.

Of the 26 regions enriched with heterozygosity in the Angus, Hereford Montana Tropical Composite, Senepol, Santa Gertrudis, and Nellore breeds [21], only the region 101.19–104.83 Mb on BTA1 of Hereford overlaps with that on BTA1 in Table 7. In the article by Mulim et al. [18], they found no identical HRRIs in sequenced Holstein bulls to those in Table 7. In Italian Simmental cattle, the HRRI on BTA21 in the range of 2.15–3.24 Mb [6] overlaps with the HRRI on BTA21 (Tables S1 and S2). Nine HRRIs identified by Ferencakovic et al. [5] in Austrian Pinzgauer cattle and the HRRI found on BTA1 in Chillingham cattle [8] do not match the HRRIs in Tables S1–S4. Thus, cattle breeds generally differ considerably in the localization of HRRIs on chromosomes. One reason for such differences may be the different density of SNPs on the SNPs arrays. For example, the 50K array cannot recognize short HRRs, partly due to their different saturation in chromosomes with an average distance between SNPs of 50 kb.

Can HRR segments identified with arrays contain homozygous SNPs? The very name “heterozygous rich regions” implies the possible presence of homozygous SNPs in these regions. Ferencakovic et al. [5] used HD arrays to identify HRRs in the genome of Austrian Pinzgau bulls. When four homozygous SNPs were allowed in the HRRs, they found 11 HRRIs, whereas using the HD array in Simmental cattle yielded no HRR segments when three homozygous SNPs were allowed [6].

Whole genome sequencing (WGS) of animals cannot yet answer this question due to the imprecise identification of homo- or hetero-SNPs, as researchers mainly use low-coverage sequencing. For example, 3–4 SNPs of the opposite genotype must be allowed in the ROH segments to allow a meaningful comparison with the array data [75]. Thanks to the advent of pangenome assemblies [76], it has become possible to accurately identify heterozygosity in HRRI islands. Further studies are required to clarify the sequencing conditions (using long and short reads) in order to obtain the most reliable SNP genotyping.

Based on the analysis of our own and published data on HRRIs, it is not possible to determine with certainty all the causes that led to their occurrence. In Holstein cattle, the effect of recombination and inversions on the formation of HRRIs is unlikely, while the presence of LINE elements may form HRRIs. The main object of balanced selection could be HRRI (66.4–66.7 Mb) on BTA1, which contains the *ILDR1*, *CD86*, and *CASR* genes and the *SYT7* gene in the HRRI (40.02–40.28 Mb) on BTA29. It can be hypothesized that some HRRI regions have arisen not only due to balancing selection, the effect of which has not yet been proven, but primarily due to the lack of inbreeding in these regions accompanied by a stochastic drifting towards final fixation or loss of alleles or structural elements.

## 5. Conclusions

Regions of high heterozygosity were identified in the genome of Holstein cows. The studied herds did not differ in the average number of HRR segments, and their lengths ranged from 0.05 to 2 Mb, much less than those of ROH segments. The number of HRRs increases when SNPs with an MAF of less than 0.01 are removed. The HRRs are unevenly distributed on the 29 chromosomes of the cows. The identification of HRRIs depends on the MAF less than 0.01 and the length of the scanned HRRs. Different inter-chromosomal HRR saturation does not cause the occurrence of HRRIs. The genes within the HRRIs are involved in immune response, reproduction, and oogenesis, but not in milk production or compositional traits. LINE elements can presumably be the cause of the occurrence of HRRIs. In summary, the phenomenon of HRRIs in Holstein cattle cannot be explained solely by recombination, long inversions, LINE elements, and balanced selection. Although any of these causes can lead to HRRIs in animals, most of these HRRIs in the Holstein breed are probably the result of a lack of long-term inbreeding effects in these chromosomal regions associated with breeding events during the selection of this breed, including the rearrangement of structural elements. However, if the Tajima D test does indeed indicate signatures of balanced selection, some HRRIs could be the result of this phenomenon, noting that balanced selection appears to be a relatively rare event in animals. It should also be noted that the Tajima D test is a necessary but not sufficient criterion for testing the signature of balanced selection.

## Figures and Tables

**Table 1:** Estimated mean (±SE) number of HRRs greater than 50 kb in the genome of Holstein cows.

Herd Number of Cows	1 N = 57	2 N = 85	3 N = 73	4 N = 44	5 N = 58	6 N = 54	The Mean Number of HRRs Across Six Herds
	Set of SNP with all MAFs						
The mean number of HRRs	337 ± 2	335 ± 1	336 ± 2	334 ± 3	333 ± 2	334 ± 3	335 ± 6
Maximum	375	382	379	382	367	376	
Minimum	300	294	292	282	296	285	
	SNPs with an MAF less than 0.01 were removed						
The mean number of HRRs	452 ± 3	436 ± 2	435 ± 2	422 ± 4	446 ± 3	442 ± 3	439 ± 7
Maximum	492	478	492	461	496	500	
Minimum	399	381	386	368	401	398	

**Table 2:** Estimated mean (±SE) number of HRRs greater than 250 kb in the genome of Holstein cows.

Herd Number of Cows	1 N = 57	2 N = 85	3 N = 73	4 N = 44	5 N = 58	6 N = 54	The Mean Number of HRRs Across Six Herds
	Set of SNP with all MAFs						
The mean number of HRRs	112 ± 1	110 ± 1	111 ± 1	111 ± 2	108 ± 1	112 ± 1	111 ± 3
Maximum	128	130	129	128	129	135	
Minimum	96	84	86	86	91	87	
	SNPs with an MAF less than 0.01 were removed						
The mean number of HRRs	205 ± 2	205 ± 2	205 ± 3	182 ± 2	202 ± 2	203 ± 2	200 ± 5
Maximum	233	228	220	209	225	237	
Minimum	173	161	166	149	176	174	

**Table 3:** Distribution of the number of HRRs greater than 50 kb by length classes in the Holstein cows.

Class (Mb)	1 N = 57	2 N = 85	3 N = 73	4 N = 44	5 N = 58	6 N = 54	The Mean Number of HRRs Across Six Herds	The Mean Proportion of HRRs
SNPs with all MAFs								
0.05–0.2	8565	13,669	11,753	7062	9289	8596	9822 ± 992	0.48
0.2–0.4	7856	12,623	10,415	6471	8517	8021	8983 ± 895	0.44
0.4–0.8	1352	2128	1913	1095	1403	1343	1539 ± 161	0.08
0.8–1.6	54	71	66	47	58	55	59 ± 4	0.003
>1.6	14	20	16	15	16	14	16 ± 1	0.0008
SNPs with an MAF < 0.01 were removed								
0.05–0.2	8594	13,651	11,742	7076	9174	8534	9795 ± 991	0.37
0.2–0.4	11,707	18,066	15,099	9050	12,810	11,767	13,083 ± 1276	0.49
0.4–0.8	3376	5019	4146	2291	3604	3314	3625 ± 372	0.13
0.8–1.6	252	333	293	146	256	235	253 ± 26	0.01
>1.6	18	24	17	17	21	17	19 ± 1	0.0007

1–6—herd numbers. N—the number of cows in a herd.

**Table 4:** Distribution of the number of HRRs greater than 250 kb by length classes in the Holstein cows.

Class (Mb)	1 N = 57	2 N = 85	3 N = 73	4 N = 44	5 N = 58	6 N = 54	The Mean Number of HRRs Across Six Herds	The Mean Proportion of HRRs
SNPs with all MAFs								
0.25–0.4	4515	7108	5965	3718	4791	4623	5120 ± 495	0.76
0.4–0.8	1352	2128	1913	1095	1403	1343	1539 ± 161	0.23
0.8–1.6	54	71	66	47	58	55	59 ± 4	0.009
>1.6	14	20	16	15	16	14	16 ± 1	0.002
SNPs with an MAF < 0.01 were removed								
0.25–0.4	7215	11,042	9240	5554	7866	7395	8052 ± 769	0.67
0.4–0.8	3376	5019	4156	2291	3604	3314	3627 ± 373	0.30
0.8–1.6	252	333	293	146	256	235	253 ± 26	0.02
>1.6	18	24	17	17	21	17	19 ± 1	0.002

1–6—herd number. N—the number of cows in a herd.

**Table 5:** Ranking of chromosomes by HRR filling, with HRRs greater than 50 kb.

**SNPs with All MAFs**										
**Chromosome**	**14**	**25**	**6**	**13**	**18**	**16**	**1**	**10**	**2**	**21**
Rank value	1.284	1.188	1.165	1.158	1.110	1.109	1.107	1.070	1.069	1.046
**Chromosome**	**7**	**19**	**8**	**28**	**3**	**17**	**15**	**23**	**5**	**26**
Rank value	1.046	1.035	1.001	0.999	0.994	0.983	0.933	0.929	0.919	0.914
**Chromosome**	**20**	**4**	**27**	**11**	**29**	**9**	**24**	**22**	**12**	
Rank value	0.900	0.899	0.891	0.888	0.883	0.860	0.804	0.803	0.797	
**SNPs with MAF < 0.01 were removed**										
**Chromosome**	**14**	**13**	**6**	**25**	**1**	**2**	**10**	**18**	**16**	**21**
Rank value	1.252	1.174	1.160	1.123	1.090	1.067	1.064	1.059	1.057	1.052
**Chromosome**	**7**	**19**	**3**	**11**	**8**	**26**	**17**	**28**	**5**	**4**
Rank value	1.021	1.015	1.000	0.993	0.980	0.966	0.960	0.949	0.946	0.932
**Chromosome**	**15**	**24**	**22**	**29**	**20**	**12**	**23**	**27**	**9**	
	0.931	0.928	0.872	0.873	0.870	0.864	0.838	0.834	0.823	

**Table 6:** Ranking of chromosomes by HRRs filling, with HRR greater than 250 kb.

**SNPs with All MAFs**										
**Chromosome**	**14**	**25**	**5**	**1**	**7**	**21**	**2**	**23**	**16**	**13**
Rank value	1.541	1.287	1.218	1.133	1.130	1.122	1.095	1.093	1.067	1.053
**Chromosome**	**18**	**9**	**6**	**19**	**3**	**29**	**8**	**4**	**17**	**10**
Rank value	1.049	1.016	1.007	1.005	0.993	0.992	0.957	0.941	0.895	0.886
**Chromosome**	**27**	**15**	**11**	**26**	**28**	**24**	**20**	**12**	**22**	
Rank value	0.880	0.842	0.837	0.835	0.829	0.82	0.768	0.745	0.584	
**SNPs with an MAF < 0.01 were removed**										
**Chromosome**	**14**	**5**	**13**	**21**	**1**	**7**	**25**	**2**	**10**	**6**
Rank value	1.405	1.249	1.157	1.135	1.121	1.116	1.106	1.095	1.048	1.034
**Chromosome**	**16**	**3**	**4**	**18**	**11**	**8**	**9**	**19**	**26**	**17**
Rank value	1.025	1.016	0.990	0.962	0.960	0.950	0.937	0.925	0.923	0.915
**Chromosome**	**29**	**22**	**24**	**15**	**12**	**23**	**28**	**20**	**27**	
Rank value	0.902	0.893	0.892	0.824	0.808	0.806	0.755	0.727	0.710	

**Table 7:** Significant HRRIs in the genome of Holstein cows.

BTA (Herd)	HRRI Regions (bp)	Number of SNPs	Length of HRRI (kb)	Proportion of HRRs	Permuted Data Sets						Mean	Mann–Whitney U Test (*p* Value)	Proportion of HRRs Across Six Herds
					1	2	3	4	5	6			
The minimum length of scanned HRRs was 50 kb, SNPs with all MAF were used													
1 (1)	66,483,743–66,668,755	4	187.0	0.62	0.46	0.40	0.52	0.50	0.54	0.48	0.48 ± 0.02	0.002	0.48
1 (2)	66,483,743–66,630,647	4	146.9	0.54	0.46	0.40	0.52	0.50	0.54	0.48	0.48 ± 0.03	0.015	0.48
1 (4)	66,483,743–66,630,647	4	146.9	0.56	0.46	0.40	0.52	0.50	0.54	0.48	0.48 ± 0.02	0.002	0.48
10 (1)	45,465,423–45,564,676	5	99.3	0.60	0.46	0.52	0.60	0.50	0.44	0.54	0.51 ± 0.02	0.015	0.50
20 (3)	40,831,029–40,986,540	4	155.5	0.62		0.52		0.50	0.58	0.56	0.54 ± 0.02	0.029	0.50
21 (4)	2,938,326–2,985,827	2	47.5	0.56			0.54	0.54	0.48	0.48	0.51 ± 0.02	0.029	0.50
The minimum length of scanned HRRs was 50 kb, SNPs with MAF < 0.01 were removed													
1 (1)	66,483,743–66,668,755	4	185.0	0.62	0.48	0.48	0.50	0.48	0.48	0.48	0.483 ± 0.003	0.002	0.46
1 (2)	66,483,743–66,630,647	3	146.9	0.54	0.48	0.48	0.50	0.48	0.48	0.48	0.483 ± 0.003	0.002	0.46
1 (4)	66,483,743–66,630,647	4	146.9	0.56	0.48	0.48	0.50	0.48	0.48	0.48	0.483 ± 0.003	0.002	0.46
1 (1)	103,675,933–103,728,420	2	52.5	0.50	0.48	0.44	0.40	0.38	0.40	0.48	0.43 ± 0.018	0.002	0.44
10 (1)	45,465,423–45,564,676	5	99.3	0.60	0.50	0.48	0.48	0.42	0.56	0.58	0.50 ± 0.02	0.002	0.50
10 (4)	45,465,423–45,564,676	5	99.3	0.58	0.50	0.48	0.48	0.42	0.56	0.58	0.50 ± 0.02	0.015	0.50
The minimum length of scanned HRRs was 250 kb, SNPs with all MAF were used													
29 (5)	40,025,469–40,281,016	4	255.5	0.54		0.42	0.40	0.46		0.50	0.44 ± 0.02	0.029	0.42
9 (5)	43,945,908–44,323,878	4	378.0	0.48	0.44		0.46		0.42	0.38	0.42 ± 0.02	0.029	
The minimum length of scanned HRRs was 250 kb, SNPs with MAF < 0.01 were removed													
29 (1)	40,025,469–40,227,347	4	202.0	0.48	0.44	0.40	0.46	0.36	0.42	0.42	0.42 ± 0.01	0.002	0.44
29 (4)	40,025,469–40,227,347	4	202.0	0.46	0.44	0.40	0.46	0.36	0.42	0.42	0.42 ± 0.01	0.015	0.44
29 (5)	40,025,469–40,227,347	4	202.0	0.54	0.44	0.40	0.46	0.36	0.42	0.42	0.42 ± 0.01	0.002	0.44
11 (2)	34,205,857–34,482,175	4	276.3	0.42	0.38	0.38	0.40	0.38			0.390 ± 0.005	0.029	0.34

**Table 8:** Tajima D values for significant HRRIs in herds of Holstein cows.

BTA (Herd)	Tajima D Regions (Mb)	Number of SNPs in the Regions	Tajima D Values	3 SD Values *	Number of Regions Exceeding 3 SD	Maximum D Value
1 (1)	66.5–66.6 65.4–66.5	4 1	3.47 1.86	2.61	114	3.47
1 (1)	103.6–103.7	3	1.95	2.61	29	3.20
9 (5)	43.9–44.0	4	2.43	2.64	20	3.25
10 (1)	45.3–45.4 45.4–45.5	3 3	2.82 2.78	2.61	10	3.09
11 (2)	34.1–34.2 34.3–34.4	2 2	2.27 2.21	2.55	48	3.22
20 (3)	40.7–40.8 40.8–40.9 40.9–41.0	3 2 3	2.93 2.51 2.49	2.88	8	3.03
21 (4)	2.8–2.9 2.9–3.0	3 3	2.83 2.52	2.59	8	2.96
29 (1)	40.0–40.1 40.1–40.2 40.2–40.3	2 2 3	2.49 2.49 2.70	2.47	23	2.91

*—Standard Deviation (SD).

**Table 9:** Mean (±SE) values of the length fraction of structural elements in 1 kb of HRRIs and ROH islands (initial data in Tables S5 and S6).

LINE (HRR islands)	0.870 ± 0.034	Mann–Whitney U test *p* = 0.011
LINE (ROH islands)	0.725 ± 0.025	
SINE (HRR islands)	0.749 ± 0.049	Mann–Whitney U test *p* = 1.0
SINE (ROH islands)	0.765 ± 0.043	
Simple repeats (HRR islands)	0.163 ± 0.020	Mann–Whitney U test *p* = 0.613
Simple repeats (ROH islands)	0.183 ± 0.024	
LTR (HRR islands)	0.216 ± 0.042	Mann–Whitney U test *p* = 0.867
LTR (ROH islands)	0.188 ± 0.028	

## Data Availability

The data supporting the findings of this study are subject to restrictions on the availability of these data in depositories other than the Russian Research Institute of Farm Animal Genetics and Breeding—Branch of the L.K. Ernst Federal Research Center for Animal Husbandry depository https://vniigen.ru/animal-genotypes/, accessed on 3 August 2025.

## Supplementary Materials

The following supporting information can be downloaded at: https://www.mdpi.com/article/10.3390/ani15152320/s1, Table S1: HRR islands in the genome of Holstein cows, provided that minimum HRR length was 50 kb (SNPs with all MAFs were saved); Table S2: HRR islands in the genome of Holstein cows, provided that minimum HRR length was 50 kb (SNPs with an MAF < 0.01 were removed); Table S3: HRR islands in the genome of Holstein cows, provided that minimum HRR length was 250 kb (SNPs with all MAFs were saved); Table S4: HRR islands in the genome of Holstein cows, provided that minimum HRR length was 250 kb (SNPs with an MAF < 0.01 were removed); Table S5: Enrichment of HRRI with structural elements; Table S6: Enrichment of ROH islands with structural elements.

## Abbreviations

The following abbreviations are used in this manuscript:

SNP	Single nucleotide polymorphism
MAF	Minor allele frequency
HRR	Heterozygosity-rich region
ROH	Runs of homozygosity
HRRI	HRR island
