# Correction: Smaragdov, M. Unravelling Heterozygosity-Rich Regions in the Holstein Genome. *Animals* 2025, *15*, 2320

**DOI:** 10.3390/ani15192795

**Published:** 2025-09-25

**Authors:** Michael Smaragdov

**Affiliations:** Russian Research Institute of Farm Animal Genetics and Breeding (RRIFAGB), Branch of the L.K. Ernst Federal Science Center for Animal Husbandry, St. Petersburg 196601, Russia; mik7252@yandex.ru

Error in Figure/Table

In the original publication [1], there was a mistake in Table 7 as published.

The original article does not provide Table 7 in full. There is no data for when the minimum length of scanned HRRs was 250 kb and SNPs with all MAFs were used and when the minimum length of scanned HRRs was 250 kb and SNPs with MAF < 0.01 were removed.

The corrected Table 7 appears below. The author states that the scientific conclusions are unaffected. This correction was approved by the Academic Editor. The original publication has also been updated.

## Figures and Tables

**Table 7:** Significant HRRIs in the genome of Holstein cows.

BTA (Herd)	HRRI Regions (bp)	Number of SNPs	Length of HRRI (kb)	Proportion of HRRs	Permuted Data Sets						Mean	Mann–Whitney U Test (*p* Value)	Proportion of HRRs Across Six Herds
					1	2	3	4	5	6			
The minimum length of scanned HRRs was 50 kb, SNPs with all MAF were used													
1 (1)	66,483,743–66,668,755	4	187.0	0.62	0.46	0.40	0.52	0.50	0.54	0.48	0.48 ± 0.02	0.002	0.48
1 (2)	66,483,743–66,630,647	4	146.9	0.54	0.46	0.40	0.52	0.50	0.54	0.48	0.48 ± 0.03	0.015	0.48
1 (4)	66,483,743–66,630,647	4	146.9	0.56	0.46	0.40	0.52	0.50	0.54	0.48	0.48 ± 0.02	0.002	0.48
10 (1)	45,465,423–45,564,676	5	99.3	0.60	0.46	0.52	0.60	0.50	0.44	0.54	0.51 ± 0.02	0.015	0.50
20 (3)	40,831,029–40,986,540	4	155.5	0.62		0.52		0.50	0.58	0.56	0.54 ± 0.02	0.029	0.50
21 (4)	2,938,326–2,985,827	2	47.5	0.56			0.54	0.54	0.48	0.48	0.51 ± 0.02	0.029	0.50
The minimum length of scanned HRRs was 50 kb, SNPs with MAF < 0.01 were removed													
1 (1)	66,483,743–66,668,755	4	185.0	0.62	0.48	0.48	0.50	0.48	0.48	0.48	0.483 ± 0.003	0.002	0.46
1 (2)	66,483,743–66,630,647	3	146.9	0.54	0.48	0.48	0.50	0.48	0.48	0.48	0.483 ± 0.003	0.002	0.46
1 (4)	66,483,743–66,630,647	4	146.9	0.56	0.48	0.48	0.50	0.48	0.48	0.48	0.483 ± 0.003	0.002	0.46
1 (1)	103,675,933–103,728,420	2	52.5	0.50	0.48	0.44	0.40	0.38	0.40	0.48	0.43 ± 0.018	0.002	0.44
10 (1)	45,465,423–45,564,676	5	99.3	0.60	0.50	0.48	0.48	0.42	0.56	0.58	0.50 ± 0.02	0.002	0.50
10 (4)	45,465,423–45,564,676	5	99.3	0.58	0.50	0.48	0.48	0.42	0.56	0.58	0.50 ± 0.02	0.015	0.50
The minimum length of scanned HRRs was 250 kb, SNPs with all MAF were used													
29 (5)	40,025,469–40,281,016	4	255.5	0.54		0.42	0.40	0.46		0.50	0.44 ± 0.02	0.029	0.42
9 (5)	43,945,908–44,323,878	4	378.0	0.48	0.44		0.46		0.42	0.38	0.42 ± 0.02	0.029	
The minimum length of scanned HRRs was 250 kb, SNPs with MAF < 0.01 were removed													
29 (1)	40,025,469–40,227,347	4	202.0	0.48	0.44	0.40	0.46	0.36	0.42	0.42	0.42 ± 0.01	0.002	0.44
29 (4)	40,025,469–40,227,347	4	202.0	0.46	0.44	0.40	0.46	0.36	0.42	0.42	0.42 ± 0.01	0.015	0.44
29 (5)	40,025,469–40,227,347	4	202.0	0.54	0.44	0.40	0.46	0.36	0.42	0.42	0.42 ± 0.01	0.002	0.44
11 (2)	34,205,857–34,482,175	4	276.3	0.42	0.38	0.38	0.40	0.38			0.390 ± 0.005	0.029	0.34
