# Supplementary material for: Unravelling Heterozygosity-Rich Regions in the Holstein Genome
Source: Animals (Basel). 2025 Aug 7;15(15):2320. doi: 10.3390/ani15152320 (PMC12346053; doi:10.3390/ani15152320)
Supplement: Supplementary file 1 [file animals-15-02320-s001.zip › Table S1.pdf]

Table S1. HRRs islands in the genome of Holstein cows provided that minimum HRRs length was 50 kb (SNPs with all MAFs were saved)

BTA (Herd)	HRRI regions (bp)	Number of SNPs	Length of HRRI (kb)	Proportion of HRRs in herd	Permuted data sets						Mean	P value of Mann-Whitney U Test [D value of Tajima D test]	Proportion of HRRs across six herds
					1	2	3	4	5	6			
1 (1)	66483743-66668755	4	187.0	0.62	0.46	0.40	0.52	0.50	0.54	0.48	0.46 ± 0.02	0.002	0.48
10 (1)	45465423-45564676	5	99.3	0.60	0.46	0.52	0.60	0.50	0.44	0.54	0.51 ± 0.02	0.015	0.50
21 (1)	29448617-29603301	5	154.7	0.56				0.52	0.50	0.48	0.50 ± 0.01	0.10	0.50
5 (1)	114355659-114499520	4	143.9	0.60								Unevaluated [max]	
2 (1)	4238852-4291955	2	53.1	0.56				0.56	0.54		0.55 ± 0.01	0.67	0.46
5 (1)	76919992-77056825	5	136.8	0.56								Unevaluated [<3 SD]	
5 (1)	114290454-114499520	5	209.1	0.56								Unevaluated [max]	
16 (1)	44041273-44223723	6	182.5	0.50				0.50	0.60		0.55±0.05	0.67	
3 (1)	83806574-83952776	5	146.2	0.50								Unevaluated [>3 SD]	
11 (1)	60377158-60498991	5	121.8	0.44				0.48		0.44	0.46 ± 0.02	0.67	0.42
10 (1)	50680586-50747119	3	66.5	0.42	0.48				0.38		0.43 ± 0.05	1.00	
14 (1)	11736525-11931937	6	195.4	0.42				0.46	0.42		0.44 ± 0.02	0.67	0.42
1 (2)	66483743-66630647	4	146.9	0.54	0.46	0.40	0.52	0.50	0.54	0.48	0.46 ± 0.03	0.015	0.48
10 (2)	45465423-45564676	4	99.3	0.54	0.46	0.52	0.60	0.50	0.44	0.54	0.51 ± 0.02	0.18 [>3 SD]	0.50
20 (2)	40852304-40986540	4	134.2	0.52		0.52		0.50	0.58	0.56	0.54 ± 0.03	1.00 [<3 SD]	0.50
6 (2)	7786578-7969025	5	182.4	0.52								Unevaluated [<3 SD]	
9 (2)	95041591-95127819	2	86.22	0.50	0.52							Unevaluated [max]	
17 (2)	59090672-59320664	5	230.0	0.48								Unevaluated [<3 SD]	
21 (2)	2918184-2985827	3	67.6	0.48			0.54	0.54	0.48	0.48	0.51 ± 0.02	0.34	
21 (2)	29448617-29563115	4	114.5	0.48				0.52	0.54	0.48	0.51 ± 0.02	0.20	0.50
2 (2)	11755807-11877444	4	121.6	0.46								Unevaluated [<3 SD]	
1 (2)	27034490-27145992	4	111.5	0.42		0.40						Unevaluated [<3 SD]	
20 (3)	40831029-40986540	4	155.5	0.62		0.52		0.50	0.58	0.56	0.54 ± 0.02	0.029	0.50
9 (3)	94991477-95065382	3	73.9	0.54	0.52	0.52					0.52 ± 0.00	0.33	
6 (3)	7786578-7969025	5	182.4	0.52								Unevaluated [>3 SD]	
9 (3)	43945908-44075848	4	130.0	0.52	0.50							Unevaluated [<3 SD]	0.48
10 (3)	45465423-45564676	5	99.3	0.48	0.48	0.52	0.60	0.50	0.44	0.54	0.51 ± 0.02	0.18	0.50
11 (3)	29231377-29326683	4	95.3	0.48		0.50		0.44	0.44		0.46 ± 0.02	0.70	
11 (3)	60377158-60498991	5	121.8	0.48				0.48		0.44	0.46 ± 0.02	0.67	0.42
16 (3)	44041273-44223723	6	182.5	0.48				0.50	0.60		0.55 ± 0.05	0.33	0.46
17 (3)	59090672-59320664	5	230.0	0.48					0.46			Unevaluated [<3 SD]	
1 (3)	52696732-52748123	3	51.4	0.46	0.48			0.42	0.38	0.42	0.42 ± 0.02	0.34	
10 (3)	80910121-80963535	3	53.4	0.44			0.48	0.42			0.45 ± 0.03	1.00	
1 (3)	52696732-52748123	3	51.4	0.40	0.48			0.42	0.38	0.42	0.42 ± 0.02	0.34	
1 (3)	66483743-66668755	5	185.0	0.40	0.46	0.40	0.52	0.50	0.54	0.48	0.48 ± 0.02	0.015*	0.48
5 (4)	51213449-51276764	2	63.3	0.60									

												Unevaluated [>3 SD]	
10 (4)	45465423-45564676	5	99.3	0.58	0.46	0.52	0.60	0.50	0.44	0.54	0.51 ± 0.02	0.065 [>3 SD]	0.50
1 (4)	66483743-66630647	4	147.0	0.56	0.46	0.40	0.52	0.50	0.54	0.48	0.48 ± 0.02	0.002	0.48
21 (4)	2938326-2985827	2	47.5	0.56			0.54	0.54	0.48	0.48	0.51 ± 0.02	0.029	0.50
16 (4)	44041273-44223723	6	182.5	0.54				0.50	0.60		0.55 ± 0.05	1.00	0.46
15 (4)	29477369-29672063	5	194.7	0.52								Unevaluated [<3 SD]	
18 (4)	39521360-39624096	5	102.7	0.48								Unevaluated [<3 SD]	
1 (4)	105191465-105293236	4	101.8	0.46	0.58	0.46			0.38	0.42	0.46 ± 0.04	0.686	0.40
11 (4)	45130713-45267174	4	136.5	0.46								Unevaluated [<3 SD]	
1 (4)	52696732-52748123	3	51.4	0.44	0.48			0.42	0.38	0.40	0.42 ± 0.02	0.343	
29 (5)	40025469-40281016	5	255.6	0.54								Unevaluated [<3 SD]	
9 (5)	94991477-95065382	4	136.4	0.52	0.52							Unevaluated [max]	
5 (5)	51213449-51392175	4	178.7	0.50								Unevaluated [max]	
14 (5)	53174026-53371542	5	197.5	0.48		0.60			0.44		0.52 ± 0.08	1.00 [max]	0.42
20 (5)	40852304-40986540	4	134.2	0.48		0.52			0.50	0.60	0.54 ± 0.03	0.100 [<3 SD]	0.50
1 (5)	128843110-128938068	5	95.0	0.44	0.38	0.40					0.39 ± 0.01	0.200	
2 (6)	4238852-4291955	2	53.1	0.58				0.56	0.58		0.57 ± 0.01	0.67	
21 (6)	29448617-29563115	3	114.5	0.54				0.52	0.54	0.48	0.51 ± 0.02	0.20	0.50
9 (6)	95041591-95127819	2	86.2	0.52	0.50	0.52					0.51 ± 0.01	0.67 [>>3 SD]	0.50
1 (6)	66483743-66668755	5	185.0	0.48	0.46	0.40	0.52	0.50	0.54	0.48	0.48 ± 0.02	0.39	0.48
10 (6)	45465423-45564676	5	99.3	0.48	0.46	0.52	0.60	0.50	0.44	0.54	0.53 ± 0.02	0.015*	0.50
14 (6)	53174026-53371542	5	197.5	0.48	0.48		0.50		0.44		0.47 ± 0.02	1.00	0,42
16 (6)	45111648-45309651	6	198.0	0.48					0.44	0.46	0.45 ± 0.01	0.33	
20 (6)	40852304-40986540	4	134.2	0.48		0.52		0.50		0.60	0.54 ± 0.03	0.10	0.50

* - Significant excess of the permuted data over the data for the herd.
