# Supplementary material for: Unravelling Heterozygosity-Rich Regions in the Holstein Genome
Source: Animals (Basel). 2025 Aug 7;15(15):2320. doi: 10.3390/ani15152320 (PMC12346053; doi:10.3390/ani15152320)
Supplement: Supplementary file 1 [file animals-15-02320-s001.zip › Table S2.pdf]

Table S2. HRRs islands in the genome of Holstein cows provided that minimum HRRs length was 50 kb (SNPs with MAF < 0.01 were removed)

BTA (Herd)	HRRI regions (bp)	Number of SNPs	Length of HRRIs (kb)	Proportion of HRRs in herd	Permuted data sets						Mean	P value of Mann-Whitney U Test [D value of Tajima D test]	Proportion of HRRs across six herds
					1	2	3	4	5	6			
1 (1)	66483743-66668755	4	185.0	0.62	0.48	0.48	0.50	0.48	0.48	0.48	0.483 ± 0.003	0.002	0.46
10 (1)	45465423-45564676	5	99.3	0.60	0.50	0.48	0.48	0.42	0.56	0.58	0.50 ± 0.02	0.002	0.50
21 (1)	2944861-29603301	5	154.7	0.56				0.52				Unevaluated [>3 SD]	0.50
5 (1)	114355659-114499520	4	143.9	0.60								Unevaluated [max]	
2 (1)	4238852-4291955	2	53.1	0.56						0.56		Unevaluated [<3 SD]	0.46
5 (1)	76919992-77056825	5	136.8	0.56		0.52						Unevaluated [<3 SD]	
10 (1)	55591993-55611885	2	53.1	0.54								Unevaluated [>3 SD]	
1 (1)	103675933-103728420	2	52.5	0.50	0.48	0.44	0.40	0.38	0.40	0.48	0.43 ± 0.018	0.002	0.44
16 (1)	44041273-44223723	6	182.5	0.50		0.48		0.58			0.53 ± 0.05	1.00	0.46
11 (1)	61858071-62067321	5	209.3	0.46		0.52		0.46		0.46	0.48 ± 0.02	0.70	0.44
1 (2)	66483743-66630647	3	146.9	0.54	0.48	0.48	0.50	0.48	0.48	0.48	0.483 ± 0.003	0.002	0.46
10 (2)	45465423-45564676	5	99.3	0.54	0.50	0.48	0.48	0.42	0.56	0.58	0.50 ± 0.02	0.39 [> 3 SD]	0.50
20 (2)	40852304-40986540	4	134.2	0.52	0.56			0.64			0.60 ± 0.04	0.33 [<3SD]	0.50
6 (2)	7786578-7969025	5	182.4	0.52			0.54					Unevaluated [>3 SD]	
9 (2)	95041591-95127819	2	86.2	0.50		0.52	0.58				0.55 ± 0.03	0.33	
17 (2)	59090672-59320664	5	230.0	0.48				0.48				Unevaluated [<3 SD]	
21 (2)	2918184-2985827	3	67.6	0.48	0.54		0.52	0.52			0.53 ± 0.01	0.10	0.50
2 (2)	11755807-11877444	4	121.6	0.46								Unevaluated [<3 SD]	
20 (3)	40831029-40986540	4	155.5	0.62	0.56			0.64			0.60 ± 0.04	1.00	0.50
6 (3)	53022829-53022829	5	182.4	0.52					0.56			Unevaluated [>3 SD]	
9 (3)	94991477-95065382	3	73.9	0.54		0.52	0.58				0.55 ± 0.03	1.00	
10 (3)	45465423-45564676	(1)5	99.3	(0.52)0.50	0.50	0.48	0.48	0.42	0.56	0.58	0.50 ± 0.02	0.70	0.50
6 (3)	7786578-7969025	5	182.4	0.52			0.54					Unevaluated [>3 SD]	
11 (3)	61968503-62067321	4	98.8	0.50				0.46		0.46		0.33	
17 (3)	59112241-59320664	4	208.4	0.50				0.48				Unevaluated [<3 SD]	
11 (3)	29231377-29326683	4	95.3	0.48		0.44				0.44		0.33	
11 (3)	60377158-60498991	5	121.8	0.48	0.44	0.52		0.44	0.46		0.47 ± 0.02	0.34	0.42
16 (3)	44041273-44223723	6	182.5	0.48		0.48		0.58			0.53 ± 0.05	0.67	0.46
1 (3)	52696732-52748123	3	51.4	0.46	0.48	0.36	0.40	0.36	0.38	0.34	0.39 ± 0.02	0.065	0.38
5 (4)	51213449-51276764	2	63.3	0.60								Unevaluated [>3 SD]	
10 (4)	45465423-45564676	5	99.3	0.58	0.50	0.48	0.48	0.42	0.56	0.58	0.50 ± 0.02	0.015 [>3 SD]	0.50
1 (4)	66483743-66630647	4	147.0	0.56	0.48	0.48	0.50	0.48	0.48	0.48	0.483 ± 0.003	0.002	0.48
21 (4)	2938326-2985827	2	47.5	0.56	0.54		0.52	0.52			0.53 ± 0.01	0.10	0.50
16 (4)	44041273-44223723	6	182.5	0.54		0.48		0.58			0.53 ± 0.05	1.00	0.46
15 (4)	29477369-29672063	5	194.7	0.52								Unevaluated [<3 SD]	
1 (4)	105191465-105293236	4	101.8	0.46	0.46							Unevaluated [>3SD]	0.40
11 (4)	45130713-45267174	4	136.5	0.46				0.44				Unevaluated [<3 SD]	
1 (5)	127426647-127546305	4	119.7	0.54		0.46			0.46		0.46 ± 0.0	0.33	0.42
9 (5)	94991477-95127819	4	136.3	0.52		0.52	0.58				0.55 ± 0.03	0.67	
9 (5)	43945908-44075848	4	130.0	0.54								Unevaluated [<3 SD]	

29 (5)	40025469-40281016	5	255.5	0.54								Unevaluated [<3 SD]	
21 (5)	2938326-2985827	2	47.5	0.50	0.54		0.52	0.52			0.53 ± 0.01	0.10	0.50
5 (5)	51213449-51392175	4	178.7	0.50								Unevaluated [>3 SD]	
14 (5)	53174026-53371542	5	197.5	0.48	0.52					0.48	0.50 ± 0.02	0.67 [max]	
20 (5)	40852304-40986540	4	134.2	0.48	0.56			0.64			0.60 ± 0.04	0.33 [<3 SD]	0.50
15 (5)	29477369-29672063	5	194.7	0.46								Unevaluated [<3 SD]	
1 (5)	105191465-105293236	4	101.8	0.44			0.46			0.38		1.00	0.40
1 (5)	128843110-128938068	5	95.0	0.44				0.44				Unevalued [<3 SD]	
11 (5)	61968503-62067321	4	98.8	0.44		0.52		0.46		0.46	0.48±0.02	0.10	0.42
2 (6)	4238852-4291955	2	53.1	0.58								Unevalued [<3 SD]	0.46
21 (6)	8231966-8312779	4	80.8	0.54		0.50						Unevalued [max]	
21 (6)	29448617-29563115	3	114.5	0.54	0.54		0.52	0.52			0.53 ± 0.01	0.20 [<3 SD]	0.50
9 (6)	95041591-95127819	2	86.2	0.52		0.52	0.58				0.55 ± 0.03	0.67 [>3 SD]	0.50
16 (6)	45229504-45309651	2	80.1	0.50						0.52		Unevalued [< 3 SD]	
1 (6)	66483743-66668755	5	185	0.48	0.48	0.48	0.50	0.48	0.48	0.48	0.483 ± 0.003	0.70	0.44
1 (6)	103623793-103728420	3	104.6	0.48	0.48	0.44	0.40	0.38	0.40	0.48	0.43 ± 0.018	0.065	0.42
10 (6)	45465423-45564676	5	99.3	0.48	0.50	0.48	0.48	0.42	0.56	0.58	0.50 ± 0.02	0.39	0.50
14 (6)	53174026-53371542	5	197.5	0.48						0.48		Unevalued[>3 SD]	
