# Supplementary material for: Unravelling Heterozygosity-Rich Regions in the Holstein Genome
Source: Animals (Basel). 2025 Aug 7;15(15):2320. doi: 10.3390/ani15152320 (PMC12346053; doi:10.3390/ani15152320)
Supplement: Supplementary file 1 [file animals-15-02320-s001.zip › Table S3.pdf]

Table S3. HRRs islands in the genome of Holstein cows provided that minimum HRRs length was 250 kb (SNPs with all MAFs were saved)

BTA (Herd)	HRRI regions (bp)	Number of SNPs	Length of HRRI (kb)	Proportion of HRRs	Permuted data sets						Mean	Mann-Whitney U Test (P value)	Proportion of HRRs across six herds
					1	2	3	4	5	6			
29 (1)	40025469-40281016	4	255.5	0.46		0.42	0.40	0.46		0.50	0.45 ± 0.02	0.68	0.42
29 (5)	40025469-40281016	4	255.5	0.54		0.42	0.40	0.46		0.50	0.45 ± 0.02	0.029	0.42
4 (2, 5)	29740103-30038994	5	300.0	0.48		0.48	0.50	0.42			0.47 ± 0.02	1.0	0.40
9 (3, 2)	94824800-95127819	5	303.0	0.48	0.52	0.48	0.46	0.46		0.38	0.46 ± 0.02	0.31	0.44
15 (2)	10373621-10700852	5	537.2	0.42			0.40					Unevaluable [<3 SD]	0.32
14 (3)	53300014-53371542	3	71.5	0.42		0.40	0.44		0.40	0.40	0.41 ± 0.01	0.34	0.38
5 (5)	51213449-51392175	4	178.7	0.48			0.46					Unevaluable [>3 SD]	
9 (5)	43945908-44323878	4	378.0	0.48	0.44		0.46		0.42	0.38	0.425 ± 0.017	0.029	0.40
