# Supplementary material for: Unravelling Heterozygosity-Rich Regions in the Holstein Genome
Source: Animals (Basel). 2025 Aug 7;15(15):2320. doi: 10.3390/ani15152320 (PMC12346053; doi:10.3390/ani15152320)
Supplement: Supplementary file 1 [file animals-15-02320-s001.zip › Table S4.pdf]

Table S4. HRRs islands in the genome of Holstein cows provided that minimum HRRs length was 250 kb (SNPs with MAF < 0.01 were removed)

BTA (Herd)	HRRI regions (bp)	Number of SNPs	Length of HRRIs (kb)	Proportion of HRRs in herd	Permuted data sets						Mean	P value of Mann-Whitney U Test [D value of Tajima D test]	Proportion of HRRs across six herds
					1	2	3	4	5	6			
29 (1)	40025469-40227347	4	202.0	0.48	0.44	0.40	0.46	0.40	0.42	0.48	0.43 ± 0.01	0.015	0.44
7 (1)	40337451-40617615	3	280.2	0.44								Unevaluated [<3 SD]	
9 (1)	4130601-4386831	6	256.2	0.40					0.40	0.46	0.43 ± 0.03	0.67	0.38
1 (1)	103973956-104344681	8	370.7	0.38	0.32	0.38	0.36	0.38		0.36	0.36 ± 0.01	0.151	0.36
11 (1)	73092085-73514952	8	422.9	0.38								Unevaluated [<3 SD]	
9 ()2	94824800-95127819	5	303.0	0.48	0.46	0.44	0.56				0.49 ± 0.04	0.70	0.44
4 (2)	29740103-30038994	5	299.0	0.48					0.56	0.48	0.52 ± 0.04	0.67	0.40
29 (2)	40025469-40227347	4	202.0	0.44	0.44		0.46	0.40	0.42	0.48	0.44 ± 0.01	1.00	0.44
11 (2)	34205857-34482175	4	276.3	0.42	0.38	0.38	0.40	0.38			0.390 ± 0.005	0.029	0.34
15 (2)	10373621-10700852	5	327.2	0.42				0.36				Unevaluated [<3 SD]	
5 (2)	119949553-120155373	4	205/8	0.42			0.46		0.40		0.43 ± 0.03	1.00	
9 (3)	94824800-95127819	5	303.0	0.48	0.46	0.44	0.56			0.42	0.47 ± 0.03	0.34 [max]	0.44
12 (3)	41666540-42005184	3	338.6	0.44		0.48	0.34	0.42	0.38		0.40 ± 0.03	0.34 [>3 SD]	0.36
14 (3)	53300014-53371542	3	75.1	0.42	0.46	0.46				0.38	0.43 ± 0.03	0.70 [>3 SD]	0.38
29 (3)	40025469-40227347	4	202.0	0.40	0.44	0.40	0.46	0.36	0.42	0.42	0.42 ± 0.01	0.18	0.44
20 (3)	41201777-41478242	7	276.5	0.40	0.38							Unevaluated [<3 SD]	
29 (4)	40025469-40227347	4	202.0	0.46	0.44	0.40	0.46	0.40	0.42	0.42	0.42 ± 0.01	0.015	0.44
5 (4)	74551645-74808483	3	256.8	0.46								Unevaluated [<3 SD]	
5 (4)	51213449-51392175	4	178.7	0.44						0.40		Unevaluated [>3 SD]	
29 (5)	40025469-40227347	4	202.0	0.54	0.44	0.40	0.46	0.36	0.42	0.42	0.42 ± 0.01	0.002	0.44
4 (5)	29740103-30038994	5	298.9	0.48					0.56	0.48	0.52 ± 0.04	0.67	0.40
5 (5)	51213449-51392175	4	178.7	0.48						0.40		Unevaluated [>3 SD]	
9 (5)	43945906-44323878	4	378.0	0.48	0.40		0.44			0.42	0.42 ± 0.01	0.10	0.40
9 (5)	94824800-95127819	5	303.0	0.44	0.46	0.44	0.56			0.42	0.47 ± 0.03	0.69	0.44
4 (6)	29740103-30038994	4	296.9	0.46					0.56	0.48	0.52 ± 0.04	1.00	0.40
9 (6)	94824800-95127819	5	303.0	0.44	0.46	0.44	0.56				0.49 ± 0.04	0.20	0.44
14 (6)	53174026-53371542	5	197.5	0.42	0.46	0.46				0.38	0.43 ± 0.03	0.70	0.38
15 (6)	10373621-10544820	3	171.2	0.42				0.36				Unevaluated [<3 SD]	
2 (6)	79846105-79946595	4	100.5	0.42						0.50		Unevaluated [<3 SD]	
