# Supplementary material for: Unravelling Heterozygosity-Rich Regions in the Holstein Genome
Source: Animals (Basel). 2025 Aug 7;15(15):2320. doi: 10.3390/ani15152320 (PMC12346053; doi:10.3390/ani15152320)
Supplement: Supplementary file 1 [file animals-15-02320-s001.zip › Table S5.pdf]

Table S5. Enrichment of HRRI with structural elements

HRRI	1:66484666 - 66668797	1:103675933 – 103728420	10:45465752 - 45564764	11:34206716 - 34482550	20:40831071 - 40987066	29:40025482 - 40227436	9:43945908 – 44323878	21:29448617 - 29603301
Number of LINE	174 (0,945)	50 (0,953)	96 (0.970)	242 (0,877)	129 (0,827)	47 (0,233)*****	300 (0,794)	113 (0,730)
Number of SINE	153 (0.831)	37 (0,705)	89 (0,899)	195 (0,707)	139 (0,891)	115 (0,569)	255 (0,675)	83 (0,537)
Number of Simple Repeats	25 (0.136)	5 (0.095)	11 (0.111)	70 (0.254)	28 (0.179)	23 (0.114)	84 (0.222)	30 (0.194)
Number of LTR	36 (0.196)	21 (0.400)	11 (0.111)	59 (0.214)	29 (0.186)	14 (0.069)	65 (0.172)	59 (0.381)
Length of HRRI (kb)	184.131	52.487	99.0	275.874	155.995	201.954	377.970	154.684

The number of elements/HRRI length is given in brackets, i.e. equal to the length fraction of structural elements in 1 kb of HRRI

. * - Outlier
