# Supplementary material for: Unravelling Heterozygosity-Rich Regions in the Holstein Genome
Source: Animals (Basel). 2025 Aug 7;15(15):2320. doi: 10.3390/ani15152320 (PMC12346053; doi:10.3390/ani15152320)
Supplement: Supplementary file 1 [file animals-15-02320-s001.zip › Table S6.pdf]

Table S6. Enrichment of ROH islands with structural elements

ROH-island	8:61614265 - 62398995	26:22118554 - 22301642	8:107707691 - 108094943	20:43589165 - 44296630	7:42440064 - 43538228	14:33964818 34100477	1:83547131 - 83643332
Number of LINE	578 (0.736)	144 (0.786)	295 (0.762)	584 (0.825)	750 (0.683)	88 (0.649)	61 (0.634)
Number of SINE	628 (0.80)	114 (0.623)	379 (0.979)	530 (0.749)	735 (0.669)	108 (0.796)	71 (0.738)
Number of Simple Repeats	105 (0.134)	37 (0.202)	114 (0.294)	170 (0.099)	198 (0.180)	29 (0.214)	15 (0.156)
Number of LTR	142 (0.181)	24 (0.131)	96 (0.248)	198 (0.280)	193 (0.176)	9 (0.066)	23 (0.239щ)
Length of ROH-island (kb)	784.730	183.088	387.252	707.465	1 098.164	135.658	96.201

The number of elements/ROH island length is given in brackets, i.e. equal to the length fraction of structural elements in 1 kb of ROH Island.
